# Use of a chromogenic medium with and without selective enrichment to screen for carbapenemase-producing *Enterobacterales* (CPE) from canine and feline fecal specimens during an outbreak of NDM-5–producing *Escherichia coli*

**DOI:** 10.1177/10406387231204560

**Published:** 2023-11-02

**Authors:** Stephen D. Cole, Jaclyn Dietrich, Shelley C. Rankin

**Affiliations:** Department of Pathobiology, School of Veterinary Medicine, University of Pennsylvania, Philadelphia, PA, USA

**Keywords:** carbapenem-resistant *Enterobacterales*, carbapenemase, companion animals, screening, selective enrichment, veterinary infection prevention

## Abstract

Carbapenemase-producing *Enterobacterales* (CPE) are one of the most urgent threats to human healthcare globally. Descriptions of CPE outbreaks in veterinary hospitals suggest the need for screening strategies for CPE from companion animals. Our aim was to optimize a chromogenic agar method with and without selective enrichment to isolate CPE from companion animal feces in an ongoing outbreak of New Delhi metallo-β-lactamse-5 *Escherichia coli*. A limit of detection (LOD) assay for spiked canine and feline feces was performed for both methods using a carbapenamase-producing *E. coli* (24213-18); the LOD (1.5 × 10^3^ cfu/g of feces) was equivalent to that reported for human fecal specimens. We screened 1,247 companion animal fecal specimens for carriage of CPE by 1) direct plating to chromogenic agar and 2) plating to chromogenic agar following selective enrichment. Twenty-one specimens were positive for CPE by both direct culture and enrichment culture. No specimens were positive with selective enrichment and negative by direct culture. A selective enrichment step did not result in any increased recovery of CPE from companion animals, which suggests that enrichment broth may not be necessary for outbreak surveillance testing. It is important to continue to validate methods for the detection of CPE in companion animals as outbreaks become more common in veterinary facilities.

The spread of carbapenemase-producing *Enterobacterales* (CPE) is one of the most urgent threats that face human healthcare globally.^16,18^ Carbapenemase enzymes produced by CPE hydrolyze carbapenems and other β-lactam drugs.^6^ Additionally, strains of CPE are often resistant to most clinically relevant antimicrobials, which makes infections caused by CPE difficult to treat and life-threatening.^6,16,18^ The 5 common carbapenemase enzyme families described in *Enterobacterales* are: *Klebsiella pneumoniae* carbapenemase (KPC), New Delhi metallo-β-lactamase (NDM), Ve-rona integron metallo-β-lactamase (VIM), imipenemase (IMP), and OXA-48-like.^18^ Metallo-β-lactamases NDM, VIM, and IMP are classified as Ambler class B. KPC and OXA-48 use serine ester hydrolysis and are classified as Ambler class A and D, respectively.^18^ CPE colonize the gastrointestinal tract of people without producing symptoms of infection, which makes CPE a particular challenge for infection control programs.^3,16,18^ Silent spread of CPE occurs between patients in hospital settings, particularly among immunosuppressed people and people receiving antimicrobial therapy.^1,3^

Most reports of CPE isolated from dogs and cats are limited to a single or a few cases,^17^ but recent descriptions of CPE outbreaks in veterinary hospitals suggest the need for strategies to respond to CPE quickly and aggressively.^2,4,10^ Strategies for control of CPE in companion animals have not been well-defined. The accurate and rapid laboratory detection of gastrointestinal colonization has been critical to the control of CPE in human hospitals during outbreaks.^1,3,16,18^ In human hospitals, rapid identification of colonized individuals allows for instituting strategies such as cohort nursing and appropriate personal protective equipment.^3^ The same is likely to be true in veterinary clinical settings; however, no microbiology laboratory methods have been evaluated for the screening of CPE in animal feces for infection control purposes, to our knowledge. A 2020 report suggested that many veterinary laboratories in the United States are underprepared to identify CPE.^20^ Veterinary diagnosticians and clinicians must take an aggressive approach to the detection of CPE in animals given that the detection of CPE is reportable in many jurisdictions. It is likely that diagnostic laboratories working with veterinary facilities in which CPE outbreaks occur may need to modify their methodology to balance costs with timely reporting of results. Molecular detection of carbapenemase genes by real-time PCR is the current gold standard for rapid detection of carbapenemase-producing CPE,^8^ but the cost and resources to perform molecular tests may limit their utility in veterinary medicine.

Chromogenic agars have been evaluated for the detection of CPE in human fecal specimens.^11,19^ However, such agars are considerably more expensive and often have a shorter shelf-life than other prepared media. Selective enrichment is often used in microbiology laboratories to promote the growth of target organisms and suppress the growth on non-target organisms from complex specimens with diverse bacterial loads such as feces.^15^ Selective enrichment is not included in the manufacturer’s protocols for the use of chromogenic media in the detection of CPE. However, selective enrichment culture steps are routinely included for the isolation of *Salmonella* spp. from canine fecal specimens^9^ and have been shown to be useful for isolation of other multidrug-resistant organisms from companion animal specimens.^7^ Our aim was to assess the use of a chromogenic agar method with and without enrichment culture to isolate CPE from companion animal feces in an ongoing outbreak of NDM-5 *Escherichia coli* at a veterinary teaching hospital.

We performed limit of detection (LOD) assays for canine and feline feces in duplicate using an isolate of *bla*_NDM-5_ *Escherichia coli* (24213-18) previously characterized as part of an ongoing outbreak.^4^ A 0.5 McFarland standard (~1.5 × 10^8^ cfu/mL) suspension of *E. coli* 24213-18 was prepared in saline, and a 1:10 dilution series was performed. A 1-mL aliquot of each dilution (10^1^–10^7^) was mixed thoroughly with 2 samples of feces (1 g each; 1 sample obtained from a dog and the other from a cat, with both animals previously determined to be free of CPE). A sterile swab (Specimen collection device; Cepheid) was dipped once into the spiked feces and used to inoculate a chromogenic agar plate (CHROMID CARBA; bioMérieux). The plates were incubated inverted at 35 ± 2°C for 18 h under atmospheric conditions. Enrichment was performed simultaneously by inoculating a swab with the spiked feces that was placed into 5 mL of trypticase soy broth (TSB; Remel) containing a 10-µg meropenem (MEM) Kirby–Bauer disc (Sensi-Disc; BD-BBL).^11^ The inoculated TSB-MEM was incubated 35 ± 2°C for 18 h under atmospheric conditions as a selective enrichment for carbapenem-resistant organisms. A 10-µL loop of the TSB-MEM broth was then streaked to chromogenic agar and incubated as described above. A representative, presumptive positive colony (pink-to-burgundy) from the lowest positive dilution was confirmed to harbor an NDM gene by a commercial PCR (Carba-R assay; Cepheid).^8^

We also compared direct and enrichment culture on chromogenic agar. Specimens from dogs and cats were collected as part of a biosecurity program established as part of a public health response to a CPE outbreak. Fecal specimens (collected either by free-catch or rectal swab) were submitted to the clinical microbiology laboratory at the Ryan Veterinary Hospital (RVH) at the University of Pennsylvania’s School of Veterinary Medicine (Philadelphia, PA, USA). Submission of diagnostic specimens is considered part of clinical care and does not require institutional approval. For the direct method, feces was inoculated onto the chromogenic agar plate per the manufacturer’s instructions. For the enrichment method, feces was inoculated into TSB-MEM broth, incubated, and subcultured to chromogenic agar as described above for the LOD assay. Plates were examined after 18–24 h of incubation for pink-to-burgundy-colored colonies (presumptive *E. coli*) or blue-gray or purple colonies (presumptive *Klebsiella*, *Enterobacter*, *Serratia*, or *Citrobacter* spp.). Presumptive CPE were subcultured to MacConkey agar (Remel) and identified with the GN identification card on the Vitek 2 (bioMérieux). Production of a carbapenemase enzyme was confirmed by the modified carbapenem inactivation method (mCIM),^14^ the minimum inhibitory concentration for imipenem was determined by E-test (bioMérieux), and the carbapenemase gene was identified with the Carba-R assay on the GeneXpert (Cepheid), which tests for carbapenemase genes (*bla*_KPC_, *bla*_NDM_, *bla*_VIM_, *bla*_IMP_, *bla*_OXA-48_).^8^

The LOD following direct plating to chromogenic agar was 1.5 × 10^3^ cfu/g of feces for both canine and feline feces spiked with the 24213-18 isolate. Following the enrichment culture step (TSB-MEM broth), the LOD was 1.5 × 10^2^ cfu/g of cat feces and 1.5 × 10^3^ cfu/g of dog feces. Between July 2019 and March 2021, we screened 1,247 animal specimens (979 canine and 244 feline) for carriage of CPE by direct plating to chromogenic agar and by plating to chromogenic agar following the selective enrichment culture described above. A total of 21 specimens (1.6%), 18 from dogs and 3 from cats, were positive for CPE by direct culture to a chromogenic agar plate (Table 1). Twenty isolates were identified as *E. coli* and one as *E. cloacae* complex. All presumptive isolates were positive for carbapenemase production by mCIM. Twenty isolates (19 *E. coli* and 1 *E. cloacae*) were positive for a *bla*_NDM_ gene and 1 *E. coli* isolate was positive for a *bla*_KPC_ gene. All corresponding selective enrichment cultures were also positive, and the same carbapenemase gene was identified. No specimens were positive by selective enrichment and negative by direct culture.

**Table 1.** Summary of carbapenemase-producing *Enterobacterales* (CPE) isolated on chromogenic agar, with and without selective enrichment, from animal feces.

Organism/Host species	No. of isolates	Imipenem MIC, µg/mL	Carbapenemase detected
*Escherichia coli*			
Cat	1	4	NDM
	1	8	NDM
	1	16	NDM
Dog	2	4	NDM
	9	8	NDM
	5	16	NDM
	1	16	KPC
*Enterobacter cloacae*			
Dog	1	16	NDM

KPC = *Klebsiella pneumoniae* carbapenemase; NDM = New Delhi metallo-β-lactamase.

A direct plate method is consistent with the manufacturer’s instructions and has been shown to be a sensitive method (> 90%) compared to the recommended U.S. Centers for Disease Control and Prevention method with TSB-MEM.^11^ In some human studies,^5,13^ the selective enrichment method paired with chromogenic medium has been shown to identify additional positive specimens. However, in our study of over 1,200 animal fecal specimens, we found complete correlation between results of a direct chromogenic agar plate method and chromogenic agar culture following a TSB-MEM enrichment step. This suggests that a selective enrichment step may not be necessary to screen companion animals for carriage of CPE. During this outbreak, presumptive positive isolates were reported to clinicians within 24 h of receipt of the specimen, and appropriate infection control measures were implemented if the specimen was positive. Control measures at the RVH included contact precautions, isolation, enhanced environmental disinfection and monitoring, and repeated testing of positive animals at future times. The improved turnaround time may not hold true for all CPE, and continued evaluation of methodologies is needed if outbreaks of CPE are identified in companion animal facilities in the future.

Although the use of the enrichment broth improved the LOD for spiked cat feces by 10-fold (1.5 × 10^2^), both direct and enrichment methods for 244 cat specimens yielded identical results (3 positives, 241 negatives). Hence, although the improved LOD with enrichment did not aid in the detection of additional cases during this outbreak, the utility of enrichment could be considered in future surveillance efforts for cats with lower colonization burdens. It is important to acknowledge that important species differences may exist and, given the lower number of positive cats and screened specimens relative to dogs, additional evaluation of feline specimens may be necessary.

Our study was performed during an outbreak of NDM-5–producing *E. coli*. Therefore, the clonal relationship of many of the isolates is a potential limitation for broader application. However, our study lays a foundation for veterinary laboratories to start when faced with specimens to screen in a CPE outbreak. Our described method detected *E. coli* with a *bla*_NDM_ gene from the feces of 18 animals, *E. coli* with a *bla*_KPC_ gene from 1 animal, and an *E. cloacae* isolate with a *bla*_NDM_ gene from 1 animal. Even though we assessed the method with over 1,200 specimens, the relatively low prevalence of CPE in animals means that more studies on methodology are needed. There are many more possible combinations of bacterial species and carbapenemase genes than those detected in our study population, and this method may not be generalizable to the detection of all types of CPE in fecal specimens from companion animals. For example, it is possible that OXA-48-like–producing CPE were missed given that the tested chromogenic agar is not formulated for isolates that produce Ambler class D carbapenemases.

Several other chromogenic media are commercially available, but we did not assess them in our study. Our results should not be extrapolated to all chromogenic agars. The chromogenic agar that we used has been shown to be inferior to a laboratory-formulated medium to detect CPE isolates with a low-carbapenem MIC (imipenem < 4 ug/mL) from food animals.^12^ The chosen medium was adequate for our study because all outbreak isolates cultured from clinical specimens prior to our study grew readily on the medium and had an imipenem MIC > 4 µg/mL.^11^ If a laboratory is performing surveillance during a companion animal CPE outbreak, it would be prudent to evaluate the need for enrichment culture for the targeted strains with their medium of choice. Future studies should include the development and assessment of additional strategies to detect and respond to outbreaks of CPE in companion animal facilities rapidly and cost-effectively.
